# Medical Nutrition Therapy Provided by Dietitians is Effective and Saves Healthcare Costs in the Management of Adults with Dyslipidemia

**DOI:** 10.1007/s11883-023-01096-0

**Published:** 2023-05-11

**Authors:** Geeta Sikand, Deepa Handu, Mary Rozga, Desiree de Waal, Nathan D. Wong

**Affiliations:** 1 Division of Cardiology, University of California Irvine Heart Disease Prevention Program, C-240 Medical Sciences, Irvine, CA 92697-4079 USA; 2 Evidence Analysis Center, Academy of Nutrition and Dietetics, 120 South Riverside Plaza, Suite 2190, Chicago, IL 60606-6995 USA; 3 Academy of Nutrition and Dietetics, Evidence Analysis Center, 120 South Riverside Plaza, Suite 2190, Chicago, IL 60606-6995 USA; 4 University of Vermont Medical Center, 111 Colchester Ave, Burlington, VT 05401 USA; 5 Heart Disease Prevention Program, University of California Irvine Division of Cardiology, C-240 Medical Sciences, Irvine, CA 92697-4079 USA

**Keywords:** Medical nutrition therapy, Dietary counseling, Dyslipidemia, Hyperlipidemia, Hypertriglyceridemia, Cost savings

## Abstract

**Purpose of Review:**

Referral to nutrition care providers in the USA such as registered dietitian nutritionists (RDNs) for medical nutrition therapy (MNT) remains low. We summarize research on the effectiveness of MNT provided by dietitians versus usual care in the management of adults with dyslipidemia. Improvements in lipids/lipoproteins were examined. If reported, blood pressure (BP), fasting blood glucose (FBG) glycated hemoglobin (A1c), body mass index (BMI), and cost outcomes were also examined.

**Recent Findings:**

The synthesis of three systematic reviews included thirty randomized controlled trials. Multiple MNT visits (3–6) provided by dietitians, compared with usual care, resulted in significant improvements in total cholesterol (mean range: − 4.64 to − 20.84 mg/dl), low-density lipoprotein cholesterol (mean range: − 1.55 to − 11.56 mg/dl), triglycerides (mean range: − 15.9 to − 32.55 mg/dl), SBP (mean range: − 4.7 to − 8.76 mm Hg), BMI (mean: − 0.4 kg/m2), and A1c (− 0.38%). Cost savings from MNT were attributed to a decrease in medication costs and improved quality of life years (QALY).

**Summary:**

Multiple MNT visits provided by dietitians compared with usual care improved lipids/lipoproteins, BP, A1c, weight status, and QALY with significant cost savings in adults with dyslipidemia and justify a universal nutrition policy for equitable access to MNT.

## Introduction

Cardiovascular disease (CVD) is a leading cause of death in the USA and globally [1, 2]. Recent studies indicate an association between dyslipidemia and other cardiometabolic risk factors and atherosclerotic cardiovascular disease (ASCVD) [1, 2, 3••, 4•, 5•, 6, 7]. Risk factors associated with ASCVD, such as dyslipidemia, hyperglycemia, hypertension, and overweight/obesity, are modifiable with diet and other lifestyle interventions [3••, 4•, 5•, 6, 7]. Thus, multiple healthcare organizations in the USA recommend diet and lifestyle interventions, such as medical nutrition therapy (MNT) provided by dietitians, be the foundation of treatment to improve ASCVD risk factors [3••, 4•, 5•, 6, 7]. In 2015, the National Lipid Association (NLA) published strong evidence-based recommendations for patient referral to a dietitian for MNT to manage dyslipidemia [3••]. In the USA, national guidelines from the American Heart Association (AHA)/American College of Cardiology (ACC) [4•, 5•, 6], The Obesity Society (TOS) [7], and the American Diabetes Association (ADA) [8] recommend patients be referred to a nutrition care provider such as a dietitian for MNT for the treatment of dyslipidemia [3••, 4•, 5•], hypertension [4•, 5•], overweight/obesity [4•, 5•, 6, 7], hyperglycemia [8], and type 2 diabetes (T2D) [6, 8, 9].

Dietitians provide MNT in various practice settings in the USA, such as hospitals, physician offices, private practice, and other healthcare facilities in conjunction with a multidisciplinary healthcare team. However, there is a universal lack of consistency in access to nutrition care in the USA [10]. Apart from diabetes and kidney disease, Medicare beneficiaries in the USA do not have access to MNT to manage dyslipidemia and other cardiometabolic risk factors [11•]. Thus, the aim of this narrative review is to summarize the evidence that supports the clinical and cost benefit of MNT by a dietitian to manage dyslipidemia and associated conditions. These data will assist policy makers in the USA to justify universal access to MNT provided by dietitians to manage ASCVD risk factors in the clinical setting and in the health care system.

### MNT Provided by Dietitians for Dyslipidemia and Cardiometabolic Risk Factors

**Fig. 1:**
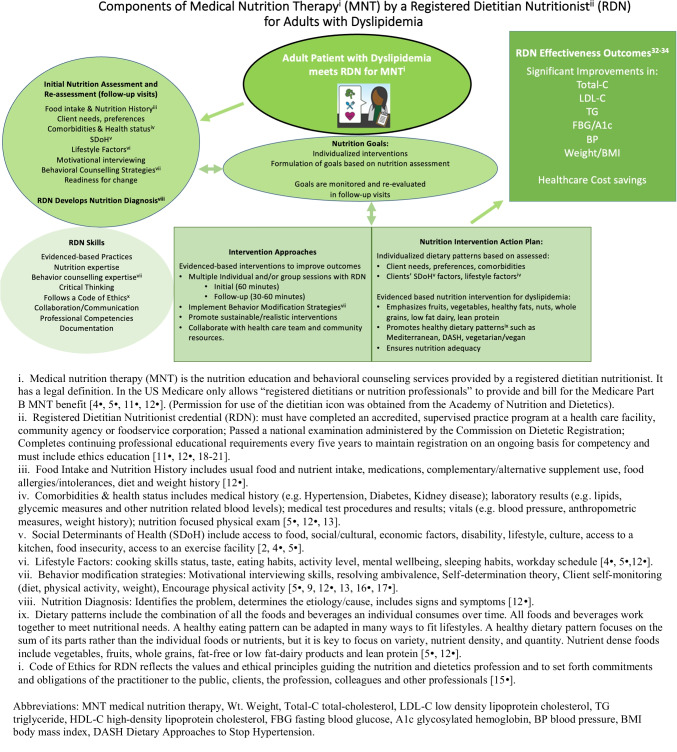
Components of medical nutrition therapy (MNT) by a registered dietitian nutritionist (RDN) for adults with dyslipidemia

MNT is an evidence-based application of the Nutrition Care Process (NCP) by the dietitian [11•, 12•, 13–15, 16•, 17•, 18–20] [Fig. 1]. MNT in the management of dyslipidemia includes nutrition assessment, nutrition diagnosis, nutrition intervention, and monitoring and evaluation to affect lipids/lipoproteins, anthropometric measures (weight, BMI, waist circumference, body fat), BP, and glycemic status with the goal to ultimately prevent or delay its cardiometabolic consequences [11•, 13, 14, 21–23]. The NCP, as defined by the Academy of Nutrition and Dietetics, helps to determine the patient’s goals as they relate to adhering to a heart-healthy dietary pattern which helps to optimize positive outcomes [11•, 12•, 13, 14, 21, 24, 25]. The dietitian incorporates evidence-based expertise in behavioral counseling methods to facilitate sustainable and desirable behavior changes [23, 26]. Dietitians collaborate with various members of the multidisciplinary health care team including physicians, nurses/nurse practitioners, exercise physiologists, physical therapists, pharmacists, and mental health professionals to provide comprehensive care [3••, 4•, 11•, 27, 28]. A multidisciplinary team approach allows each team member to enhance and complement the efforts of each other while operating at the top of their expertise [3••, 4•, 5•].

### Healthcare Cost Burden of Cardiovascular Disease and Dyslipidemia

The economic cost of CVD attributed to dyslipidemia in the USA increased from $103.5 billion in 1996 to 1997 to $226.2 billion in 2017 to 2018 [2]. The average annual direct and indirect cost of CVD in the USA was estimated at $378 billion in 2017 to 2018. By event type, hospital inpatient stays accounted for the highest direct cost ($99.6 billion) in 2017–2018 in the USA [2]. In an observational cohort study [29] of 17,183 patients comparing patients with statin-controlled LDL-C with or without high TG, the overall difference in annual costs in the high TG cohort totaled over $2.6 million per year in excess annual costs and more than $13.5 million over the mean follow-up of 5.2 years suggesting significant potential healthcare savings (e.g., per person annual savings $964 in low TG group) [29]. In a retrospective study (*n* = 193,385) to examine healthcare costs related to cardiovascular events (CVE) in individuals with hyperlipidemia, costs were highest in the acute (first 30 days) post CVE and remained high over 3 years of follow-up as compared to those without a CVE [30]. In a systematic review of the cost of illness for hypercholesterolemia and mixed dyslipidemia, the direct costs estimate as an annual expenditure ranged from $17 to $259 million [31]. The clinical and economic impact of CVE events is a well-established public health issue [31]. It seems prudent to invest in access to MNT aimed at improving lipid levels to reduce residual CVD risk which can have an impact on healthcare cost savings [29].

## Literature Review Methods

For this literature review, the population of interest was adults with dyslipidemia and the primary intervention of interest was MNT provided by dietitians. The outcomes of interest were improvements in circulating levels of lipids/lipoproteins [total-C (total cholesterol), LDL-C (low-density lipoprotein cholesterol), HDL-C (high-density lipoprotein cholesterol), TG (triglyceride)], blood pressure (BP), anthropometrics (weight, BMI), and glycemic control (fasting blood glucose (FBG) and glycated hemoglobin (A1c)]. A systematic search was performed to identify systematic reviews meeting eligibility criteria and published over the past 5 years using multiple databases (Medline (Bbsco), CINHAL (EBsco), Cochrane CENTRAL (Ebsco), and Cochrane Database of Systematic Reviews (Ebsco). Search terms included condition/population (e.g., hyperlipidemia, dyslipidemia, hypertriglyceridemia), intervention (diet therapy, medical nutrition therapy, behavior therapy, counseling, etc.), and study design (meta-analysis, systematic reviews (SR) etc.). A total of three SRs [32••, 33••, 34••] addressed the scope of this narrative review and were published within the past 5 years.

## Summary of Evidence on the Effectiveness of MNT Provided by Dietitians in the Management of Adults with Dyslipidemia

### Study and Population Characteristics

Three SRs were published in the last 5 years that evaluated the effectiveness of MNT in the management of adults with dyslipidemia [32••, 33••, 34••]. Study characteristics such as population, study designs of included studies, number and types of databases searched, sample size, risk of bias assessment, certainty of evidence, and outcomes were examined (Table 1). The three recent SRs examining the effectiveness of MNT in the management of dyslipidemia [32••, 33••, 34••] collectively included -30 unique RCTs [35–64]. Study participants also had additional ASCVD risk factors such as T2D [35, 36, 38, 39, 47, 51–53, 59, 62, 63], overweight or obesity [51, 53, 57, 60, 62], pre-diabetes [43, 53], hypertension [39, 42, 45, 47, 52, 58, 59, 61], CVD [39, 41, 59, 61], NAFLD [60], and MetS [48].

**Table 1:** Recent systematic reviews examining the effect of medical nutrition therapy in the management of adults with dyslipidemia

Systematic review	Population	Study designs included /	# of databases searched	Meta-analysis (yes/no) sample size	ROB tool	Certainty of evidence (yes/no)	Outcomes of interest reported
Mohr et al. 2022 [32••]	Adults with dyslipidemia (*n* = 838)	RCTs (*n* = 8)	4 (MEDLINE, CINAHL, Cochrane CENTRAL, and Cochrane Database of Systematic Reviews)	Yes	Cochrane’s ROB 2.0	Yes	Total-C, LDL-C, HDL-C, TG, BP
Ross et al. 2019 [34••]	Adults (≥ 18 years) at high-risk of cardiovascular disease (*n* = 1530)	RCTs (*n* = 10)	7 (ProQuest Family Health, Scopus, PubMed Central, MEDLINE, CINAHL and Cochrane)	Yes	Cochrane’s ROB	Yes	Total-C, LDL-C, HDL-C, TG
Sikand et al. 2018 [33••]	Adults with dyslipidemia and cardiovascular risk factors (*n* = 5704)	RCTs(*n* = 11) Total studies (*n* = 34) • RCTs deemed observational per research question4 • Randomized cohort studies1 • Non-randomized controlled trials2 • Non-controlled trials2 • Prospective cohorts2 • Retrospective cohorts2 • Pre-Post studies10	3 (PubMed, Medline, Worldcat.org)	Yes	Academy’s Quality Criteria Checklist	Yes	Total-C, LDL-C, HDL-C, TG, BP, FBG, A1c, BMI

*A1c*, glycosylated hemoglobin; *BMI*, body mass index; *BP*, blood pressure; *DBP*, diastolic blood pressure; *FBG*, fasting blood glucose; *HDL-C*, high-density lipoprotein cholesterol; *LDL-C*, low-density lipoprotein cholesterol; *RCTs*, randomized controlled trials; *ROB*, risk of bias; *SBP*, systolic blood pressure; *TG*, triglyceride; *Total-C*, total cholesterol

All three systematic reviews (30 RCTs) [32••, 33••, 34••] examined the effect of MNT provided by dietitians or an international equivalent (for example, in the USA, the credential is Registered Dietitian Nutritionist (RDN); in Australia the nutrition care provider’s credential is referred to as an Accredited Practicing Dietitian (APD); and in Canada, it is Registered Dietitian (RD) [65, 66].

Fifteen RCTs describe providing individual-level interventions [35, 36, 38, 46–48, 51, 53, 55–58, 60, 63, 64], and seven RCTs included group-level interventions [39, 43, 47, 49, 56, 62, 64]. Although the number of MNT sessions by the dietitians varied, most interventions included three to six sessions over three to 6 months [35–37, 39–41, 44–47, 49, 53, 54, 56, 57, 64]. Session durations varied, but typically ranged from 30 to 120 min per session [43, 44, 50, 51, 53, 54, 57, 59]. Dietary approaches, when described, varied across studies and were often based on the target population. Dietary approaches targeted MNT for hyperlipidemia [58, 64], T2D [39], population-based dietary patterns [47, 62], reduced energy [51, 57, 63], reduced fat and/or cholesterol [51, 56, 57], Mediterranean diet [55], individualized diet [57], and low-carbohydrate diets [57, 63].

### Summary of Effects on Outcomes of Interest: Circulating Levels of Lipids/Lipoproteins (Total-C, LDL-C, HDL-C, TG), BP, Anthropometrics (Weight, BMI) FBG and A1c

In the pooled analyses, all three systematic reviews (1) Mohr et al. 2022 (*n* = 838, seven RCTs)] [32••]; (2) Sikand et al. 2018 (*n* = 5704 in 34 studies/19 RCTs) [33••]; and (3) Ross et al. 2019 (*n* = 1530, 10 RCTs) [34••] reported significant improvements in lipids/lipoproteins (LDL-C, TG, total-C) with MNT provided by dietitians compared to usual care (Table 2). Furthermore, significant improvements were reported in BP [32••, 33••] and in anthropometrics (BMI), FBG, and A1c [33••]. Significant cost savings along with improved QALY and quality of life scores were also reported [33••].

- Total-C: Total-C was reduced significantly (Table 2): Mohr et al. =  − 20.84 mg/dL (*n* = 521, 4 RCTs) [32••], Sikand et al. =  − 9.9 mg/dL (*n* = 2333, 9 RCTs) [33••], and Ross et al. = non-significant reduction [34••].
- LDL-C: LDL-C was reduced significantly (Table 2): Mohr et al. =  − 11.56 mg/dL (*n* = 719, 6 RCTs) [32••], Sikand et al. = LDL-C − 10.3 mg/dL (*n* = 2526, 10 RCTs) [33••], and Ross et al. = non-significant reduction [34••].
- TG: TG was reduced significantly (Table 2): Mohr et al. =  − 32.55 mg/dL, (*n* = 620, 5 RCTs) [32••], Sikand et al. = TG levels − 15.9 mg/dL (*n* = 799, 9 RCTs) [33••], and Ross et al. =  − 19.5 mg/dL (*n* = 876, 6 RCTs) [34••].
- HDL-C: Non-significant improvements (Table 2) were reported in HDL-C in all SRs [32••, 33••, 34••].
- BP: BP improved significantly (Table 2): Mohr et al. SBP =  − 8.76 mg/dL (*n* = 208, 4 RCTs) [32••], Sikand et al. = SBP − 4.7 mm Hg (*n* = 1991, 6 RCTs), and DBP − 2.6 mm Hg (*n* = 1991, 6 RCTs), (*n* = 2526, 10 RCTs) [33••]. Ross et al. did not evaluate BP [34••].
- FBG: FBG improved significantly (Table 2): Sikand et al. =  − 5.3 mg/dL (− 9.3 to − 1.2) [*n* = 1568, 7 RCTs) [34••]. Mohr et al. [33••] and Ross et al. [35] did not evaluate FBG.
- A1c: A1c improved significantly (Table 2): Sikand et al. [23] =  − 0.38%, (*n* = 1392, 6 RCTs) [33••]. Mohr et al. [32••] and Ross et al. [34••] did not evaluate A1c.
- BMI: BMI was reduced significantly (Table 2): Sikand et al. =  − 0.4 kg/m^2^ (*n* = 1718, 8 RCTs) [33••]. Mohr et al. [32••] and Ross et al. [34••] did not evaluate anthropometrics.
- Cost savings: Cost savings of $638 to $1450 per patient per year were reported in SR 2 due to decreases in medications [33••]. Mohr et al. [33••] and Ross et al. [35] did not examine cost savings.
- QALY: SR 2 reported an increase in QALY by 0.75 years [33••]. Mohr et al. [33••] and Ross et al. [35] did not evaluate the QALY saved.

**Table 2:**
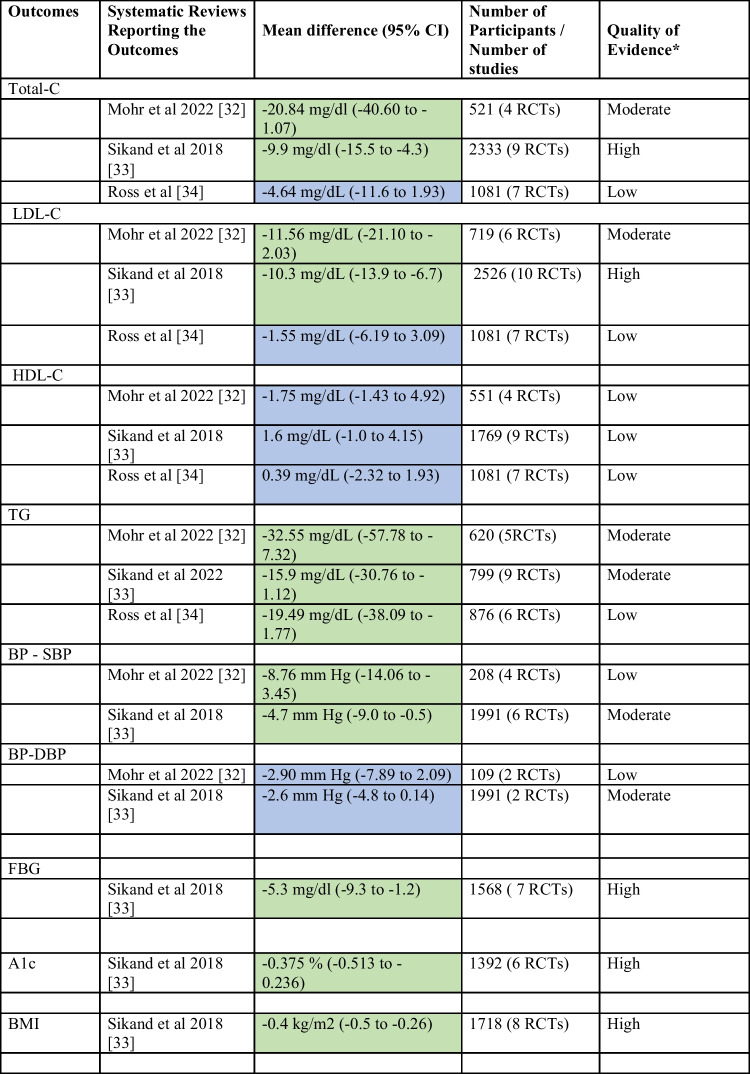
Summary of findings of the systematic review focused on the effectiveness of MNT provided by RDN for dyslipidemia

*A1c*, glycosylated hemoglobin; *BMI*, body mass index; *BP*, blood pressure; *DBP*, diastolic blood pressure; *FBG*, fasting blood glucose; *HDL-C*, high-density lipoprotein cholesterol; *LDL-C*, low-density lipoprotein cholesterol; *RCTs*, randomized controlled trials; *SBP*, systolic blood pressure; *TG*, triglyceride; *Total-C*, total cholesterol

*Certainty of evidence information was reported as stated in the included systematic reviews. These systematic reviews used GRADE method to determine certainty of evidence for outcomes of interest and graded them as high, moderate, or low

Green shading indicates significant between group effect

Blue shading indicates non-significant between group effect

## Discussion

Results from three systematic reviews consistently demonstrate the beneficial effects of MNT on dyslipidemia compared with usual care. Mohr et al. [32••] and Sikand et al. [33••] reported significant reductions in total-C, LDL-C, TG levels, and SBP and a similar non-significant increases in HDL-C, respectively. Ross et al. [34••] reported significant reductions in TG compared with usual care and reported no significant difference in total-C and LDL-C reduction between MNT and usual care (printed materials provided by a physician or nurse). This disparate finding could be attributed to its eligibility criteria [34••] which also included studies with only one face-to-face MNT visit, while Mohr et al. [32••] and Sikand et al. [33••] included studies that required at least two or more face-to-face MNT visits. Sikand et al. [33••] reported an average of three to six face-to-face MNT visits with a dietitian over 3–6 months led to significant improvements in lipids/lipoproteins, BP, FBG, A1c, and BMI suggesting that one face-to-face MNT visit is not adequate. Of note, there was little overlap between the systematic reviews due to different eligibility criteria. Out of thirty studies, only five studies (40, 43, 46, 49, 53) overlapped between the three systematic reviews. Four studies (40, 43, 46, 53) were common between Sikand et al. [33••] and Ross et al. [34••] and two studies [46, 49] between Sikand [33••] and Mohr et al. [32••].

Though the direction of findings was similar between the systematic reviews, those that had stricter inclusion criteria in relation to intervention rigor typically demonstrated greater effect sizes.

An earlier narrative review by Jacobson et al. [3••] reported significant improvements in lipids/lipoproteins (total-C, LDL-C, and TG), along with cardiometabolic risk factors (BP, A1c, and BMI) with multiple MNT visits. Another systematic review of eight studies by McCoin et al. [67] in 2008 also reported significant improvement in lipids/lipoproteins and cardiometabolic risk factors along with significant cost savings.

Although HDL-C is not considered a direct target of lifestyle therapy [3••], low HDL-C (< 35 mg/dL) levels are a strong predictor of CVD event risk. The clinical relevance of raising HDL-C with lifestyle remains uncertain and clinical trials involving medications that raise HDL-C (e.g., niacin and fibrates) have not been shown to improve CVD outcomes [3••, 4•, 68•]. The current targets of lifestyle and drug therapies are circulating levels of atherogenic cholesterol (LDL-C and non-HDL-C), and significant reductions in LDL-C were noted in all three systematic reviews [32••, 33••, 34••]. Non-HDL-C contains both LDL-C and very-low-density lipoprotein cholesterol (VLDL-C). Of note, TG levels (highly correlate with VLDL-C) were significantly improved with MNT intervention in all three systematic reviews [32••, 33••, 34••].

### Use of Lipid Lowering Medications

Of note, some participants in included the RCTs were taking lipid-lowering medications [35, 39, 41, 47, 52, 53, 58–60]. Statins remain the primary evidence-based pharmacologic strategy for treating dyslipidemia [68•]. However, the additive effect of MNT could reduce the need and dosage of medications, augment the effect of pharmacologic treatment, reduce the cost of treatment (especially if branded non-statin therapy would otherwise be needed), and reduce the residual risk of CVD [32••, 33••, 34••, 68•]. For example, Orazio [52] noted that a multidisciplinary team inclusive of pharmacological treatment can lead to improvements in cardiovascular risk factors such as lipids/lipoproteins. In Adachi et al. [35] study, medication usage was controlled as the changes in medications were not significant between the Intervention group and control group. Furthermore, Sikand [33••] reported a significant reduction in LDL-C with combining MNT with lipid-lowering medications in two RCTs [*n* = 872] [59, 69] and concluded that although the effect of MNT by dietitians may not be discerned when combined with lipid-lowering medications, these studies demonstrate that treatment goals can be achieved when MNT provided by dietitians is combined with pharmacological approach [33••]. The use of statins and anti-diabetic and BP medications may also improve some lipids, blood glucose, and BP outcomes in conjunction with MNT [33••].

### Cost Savings of MNT Provided by Dietitians for Dyslipidemia

Improving lifestyle factors, which includes nutrition, has been shown to improve outcomes for heart disease risk [3••, 4•, 5•, 6, 7, 68•, 70]. Dietitian-delivered MNT promotes changes in dietary intake of fat and saturated fat and positively impacts changes in serum lipid levels [32••, 34••, 67, 71]. When patients attended two to four MNT sessions over 6 to 12 weeks, they reduced daily dietary fat (5% to 8% saturated fat (2% to 4%) and energy intake (232–710 kcal per day). Total-C was lowered by 7% to 21%, and LDL-C was lowered by 7% to 22%. TGs were lowered from 11 to 31% [71]. Sikand et al. [33••] reported an annual cost savings of $638 to $1456 per patient from reduced medication use and a gain of 0.75 QALY per patient. In a study investigating behavioral economics to improve CVD health behaviors and outcomes, it was found that using a variety of platforms that encouraged more frequent interaction between patients and healthcare providers helped engage patients and improve their decision-making about their health [72]. Another approach using home-delivered healthy medically tailored meals (MTMs) prepared for individuals with chronic disease was associated with approximately 1.6 million averted hospitalizations and a net cost savings of about $13.6 billion annually [73]. Suboptimal diets are associated with heart disease, stroke, and T2D in the USA [74]. There is an increased interest in improving nutrition care and the availability of nutrition programs that also include medically tailored meals in the philosophy of “food is medicine” as potential tools to improve health outcomes and food security [73].

### Why Are Dietitians Successful in Assisting Individuals with Making Nutrition and Lifestyle Behavioral Changes?

Beyond their nutrition expertise, another reason dietitians achieve significant lipid-lowering effects is that they arrange for help their patients need to make behavior changes. For dietitians, the 5A model (assess, advise, agree, assist, and arrange) serves as a clinical framework for helping patients’ lifestyle behavioral changes while ensuring the individual’s autonomy [16•, 17•, 23, 26]. Thus, dietitians focus on the common barriers to lifestyle change often attributed to SDoH [16•, 17•, 23, 26]. The 5 A framework helps patients resolve their ambivalence about making lifestyle behavior changes [16•, 17•, 23, 26]. Importantly, dietitians are skilled in helping patients deal with barriers to change including ambivalence to change and also helping patients address other barriers they have, so as to make healthy lifestyle changes (examples include support at home, work environment, financial) [8, 16•, 17•, 23, 26]. In fact, in terms of the 5 As a model of change, dietitians provide support to patients by assisting them to achieve their lifestyle goals and arranging for help they need to change their behaviors. Physicians are skilled at the first three components of the 5 As the model (i.e., assessment, agreeing, and advising), but are not trained (nor have time) to assist patients and arrange for the help they need to achieve their goals [8]. In contrast, dietitians have the knowledge and expertise to do this; the systematic reviews conducted to date clearly show the benefits of MNT in helping patients achieve their lifestyle (and clinical) goals [32••, 33••, 34••]. Thus, as integral members of the health care team because of their unique expertise, dietitians contribute importantly to optimizing patient care and improving patient outcomes [3••, 32••, 33••, 34••].

### Policy Making in the USA for Universal Access to MNT Provided by Dietitians

MNT should be seamlessly integrated into clinical practice and into the USA healthcare system for equity in nutrition care to mitigate ASCVD risk factors. CVD remains the leading cause of mortality in the USA and globally [1, 2]. Dyslipidemia along with cardiometabolic risk factors, such as hyperglycemia, hypertension, and overweight/obesity, are modifiable with diet and other lifestyle interventions [32••, 33••, 34••]. MNT in the USA includes an evidence-based diet and lifestyle intervention typically provided by dietitians [11•, 13, 14, 21]. In the USA, despite strong evidence and recommendations from the NLA [3••], AHA/ACC [4•, 5•, 6], TOS [7] and ADA [8, 9], referral for MNT by dietitians remains low [10] due to a lack of universal access to nutrition care for patients with dyslipidemia, overweight/obesity, hypertension, and hyperglycemia. In addition, healthcare providers may be unaware of how to access the services of dietitians and other nutrition resources at the local, state, or national level. In the USA, healthcare providers can find dietitians by specialty and zip code through the Academy of Nutrition and Dietetics [75••]. In the USA, the policymakers of health systems should include dietitians in clinical practice and on the multidisciplinary health care teams. Improving access to nutrition care will allow healthcare providers to partner with dietitians for achieving successful outcomes in adults with dyslipidemia along with improving BP, FBG, A1c, and BMI, in conjunction with, and without lipid-lowering medications. Several resources are available for billing for nutrition services [76–79]. The expansion of MNT legislation for payment for services in the USA is a necessary step to provide nutrition care to seniors and to close the gaps in health disparities.

## Conclusion

Multiple MNT visits provided by dietitians are effective and cost beneficial in improving dyslipidemia and cardiometabolic risk factors such as overweight/obesity, elevated BP, and A1c compared to usual care. High-quality evidence from three recent SRs offers strong justification for increasing access to MNT care for dyslipidemia, overweight/obesity, hypertension, and hyperglycemia to mitigate the risks of ASCVD in conjunction with and/or without lipid-lowering medications. Treatment goals can be achieved when MNT provided by a DIETITIAN is combined with a pharmacological approach with significant cost savings. MNT is an evidence-based, cost-effective component of treatment to help combat the most prevalent and costly chronic conditions, including conditions contributing to poor COVID-19 outcomes. Improvement of efforts to increase awareness among healthcare providers in the USA on how to access dietitians and other nutritional services and resources is needed. Access to MNT is critical for seniors and communities of color that suffer from chronic disease health disparities, driven by reduced access to medical and nutrition care, healthy foods, and safe places to be active.

In the USA, MNT by dietitians should be considered for reimbursement in the treatment of dyslipidemia, hyperglycemia, hypertension, and overweight/obesity as a standard of practice to optimize cardiovascular outcomes. The expansion of MNT legislation in the USA with advocacy from major influential medical societies is necessary to provide nutrition care to seniors and to close the gaps in health disparities. Further research is needed to include longer intervention periods.
